# The Feasibility of Semi-Continuous and Multi-Frequency Thoracic Bioimpedance Measurements by a Wearable Device during Fluid Changes in Hemodialysis Patients

**DOI:** 10.3390/s24061890

**Published:** 2024-03-15

**Authors:** Melanie K. Schoutteten, Lucas Lindeboom, Hélène De Cannière, Zoë Pieters, Liesbeth Bruckers, Astrid D. H. Brys, Patrick van der Heijden, Bart De Moor, Jacques Peeters, Chris Van Hoof, Willemijn Groenendaal, Jeroen P. Kooman, Pieter M. Vandervoort

**Affiliations:** 1 Limburg Clinical Research Center/Mobile Health Unit, Faculty of Biomedical and Life Sciences, Hasselt University, Agoralaan, 3590 Diepenbeek, Belgium; helene.decanniere@uhasselt.be (H.D.C.); bart.demoor@uhasselt.be (B.D.M.);; 2 Ziekenhuis Oost Limburg, Department of Future Health, Ziekenhuis Oost Limburg Genk, Synaps Park 1, 3600 Genk, Belgium; 3 Department of Health Research, imec the Netherlands/Holst Centre, High Tech Campus 31, 5656 AE Eindhoven, The Netherlands; lucas.lindeboom@imec.nl (L.L.);; 4 Data Science Institute, Hasselt University, 3500 Hasselt, Belgium; 5 Division of Geriatrics, Department of Internal Medicine, Maastricht University Medical Centre+, P. Debyelaan 25, 6202 AZ Maastricht, The Netherlands; 6 Department of Nephrology, Jessa Ziekenhuis, Stadsomvaart 1, 3500 Hasselt, Belgium; 7 Department of Nephrology, Ziekenhuis Oost Limburg, Synaps Park 1, 3600 Genk, Belgium; 8 Imec Belgium, Kapeldreef 75, 3001 Leuven, Belgium; 9 Department of Electrical Engineering (ESAT), Katholieke Universiteit Leuven, Kasteelpark Arenberg 10, 3001 Leuven, Belgium; 10 Division of Nephrology, Department of Internal Medicine, Maastricht University Medical Centre+, P. Debyelaan 25, 6202 AZ Maastricht, The Netherlands; 11 NUTRIM School of Nutrition and Translational Research in Metabolism, Maastricht University, Universiteitssingel 40, 6229 ER Maastricht, The Netherlands; 12 Department of Cardiology, Ziekenhuis Oost Limburg, Synaps Park 1, 3600 Genk, Belgium

**Keywords:** bioimpedance, thoracic, semi continuous, multi-frequency, wearable

## Abstract

Repeated single-point measurements of thoracic bioimpedance at a single (low) frequency are strongly related to fluid changes during hemodialysis. Extension to semi-continuous measurements may provide longitudinal details in the time pattern of the bioimpedance signal, and multi-frequency measurements may add in-depth information on the distribution between intra- and extracellular fluid. This study aimed to investigate the feasibility of semi-continuous multi-frequency thoracic bioimpedance measurements by a wearable device in hemodialysis patients. Therefore, thoracic bioimpedance was recorded semi-continuously (i.e., every ten minutes) at nine frequencies (8–160 kHz) in 68 patients during two consecutive hemodialysis sessions, complemented by a single-point measurement at home in-between both sessions. On average, the resistance signals increased during both hemodialysis sessions and decreased during the interdialytic interval. The increase during dialysis was larger at 8 kHz (∆ 32.6 Ω during session 1 and ∆ 10 Ω during session 2), compared to 160 kHz (∆ 29.5 Ω during session 1 and ∆ 5.1 Ω during session 2). Whereas the resistance at 8 kHz showed a linear time pattern, the evolution of the resistance at 160 kHz was significantly different (*p* < 0.0001). Measuring bioimpedance semi-continuously and w*i*th a multi-frequency current is a major step forward in the understanding of fluid dynamics in hemodialysis patients. This study paves the road towards remote fluid monitoring.

## 1. Introduction

In many disciplines of life science, bioimpedance is known as a non-invasive and easy-to-use method for the assessment of body composition and fluid status of the human body [1,2]. Recent innovations in the field of micro-electronics have led to the miniaturization of bioimpedance devices accompanied by a longer lifespan of batteries [3]. This evolution enabled the development of wearable devices that can record bioimpedance signals semi-continuously. The thoracic region is a body-segment of specific interest for measuring bioimpedance because of the presence of the heart, lungs, and large blood vessels which determine central hemodynamic changes (i.e., blood pressure and fluid changes) [4,5,6,7,8].

Patients treated w*i*th hemodialysis are exposed to frequent fluid changes which contribute to their high cardiovascular morbidity and mortality [9]. To avoid low blood pressure during hemodialysis and fluid overload during the interdialytic interval, monitoring fluid dynamics such as changes in blood pressure and fluid volume is highly needed. Nowadays, clinicians rely on estimations of fluid status obtained by interdialytic weight gain or by a single-point (pre-dialytic), whole-body bioimpedance measurement. In addition, fluid dynamics during hemodialysis can be monitored by estimating changes in intravascular volume. However, these methods’ feasibility and accuracy remain subject to debate [10,11,12,13]. During the interdialytic interval, the use of hemodynamic monitoring is very limited [14]. Given that wearable devices have the theoretical ability to measure bioimpedance semi-continuously, the thoracic bioimpedance technique emerges as an alternative to longitudinally monitor fluid dynamics in hemodialysis patients [3,15].

The use of a wearable device measuring thoracic bioimpedance in relation to hemodynamic parameters of dialysis patients has been investigated before [5,16,17]. In these studies, repeated single-point thoracic bioimpedance measurements (i.e., pre-dialysis, every half hour or every hour during dialysis, and post-dialysis) at a single frequency (8 kHz) already showed their potential to accurately track fluid changes during hemodialysis [5,16]. Moreover, changes in thoracic bioimpedance were moderately related to changes in systolic blood pressure [18]. However, an extension to semi-continuous thoracic recordings (i.e., every ten minutes), both during dialysis and during the interdialytic interval, may offer a more detailed look into the longitudinal time pattern of the bioimpedance signal. Hence, semi-continuous recordings could predict clinical endpoints, such as dialysis-related hypotension or acute pulmonary oedema during the interdialytic interval, through longitudinal and remote hemodynamic monitoring during fluid shifts. Moreover, it is known that multi-frequency bioimpedance signals are more accurate in determining fluid volumes compared to single-frequency signals [19,20]. As such, introducing a multi-frequency electrical current to the wearable device may provide in-depth physiological knowledge on fluid changes w*i*thin and between compartments. To date, semi-continuous measurements of multi-frequency bioimpedance by a wearable device are lacking. Therefore, this study aimed to investigate the feasibility of a wearable device measuring semi-continuous multi-frequency thoracic bioimpedance in hemodialysis patients. We hypothesized we would observe a different (non-linear) time pattern in bioimpedance signals obtained by a few single-point versus semi-continuous measurements; and a different time pattern in low- versus high-frequency thoracic bioimpedance signals obtained by semi-continuous recordings.

## 2. Methods

### 2.1. Study Design

**Figure 1:**
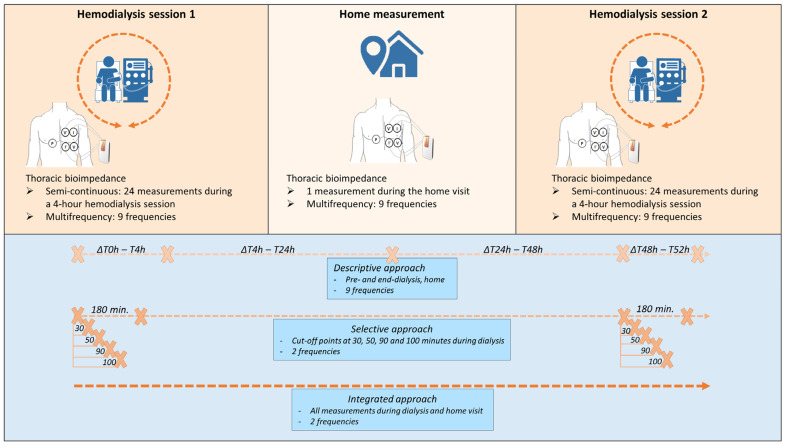
Schematic presentation of the study design and the 3 methodological approaches. I current, P bias polar, V voltage.

This study was designed as a multicenter prospective cohort and was conducted in the dialysis units of the tertiary care centers Ziekenhuis Oost-Limburg (Genk, Belgium) and Jessa Ziekenhuis (Hasselt, Belgium). Hemodialysis patients who were over 18 years old and able to provide informed consent were eligible to participate. Limb amputation, and the need for acute hemodialysis or a long-interval dialysis session were the exclusion criteria. Each patient underwent thoracic bioimpedance measurements during three consecutive days. On the first day, thoracic bioimpedance was recorded semi-continuously (i.e., every 10 min) during a 4 h hemodialysis session (Figure 1). The second day was the interdialytic day. Patients were visited at home and one single-point measurement was obtained. On the third day, which was the following hemodialysis day, the exact same measurement protocol was applied as during the first hemodialysis.

Prior to study enrollment, written informed consent was obtained from each patient. This study complies w*i*th the Declaration of Helsinki and the study protocol was approved by the local committees on human research (eudract/B-number B371201628917) of Ziekenhuis Oost-Limburg (Genk, Belgium), Jessa Ziekenhuis (Hasselt, Belgium), and Hasselt University (Hasselt, Belgium).

### 2.2. Data Collection

Patients’ medical history and dialysis data were collected from electronic medical records. Hemodialysis prescriptions were checked to determine number of sessions per week, duration of treatment, patients’ target weight, and dialysis efficacy, expressed as standard Kt/V. Patients were weighed before and after each dialysis session, and during the home-visit. As in standard clinical practice, net ultrafiltration volume was derived from interdialytic weight gain and adjusted by the treating nephrologist based on clinical examination.

**Figure 2:**
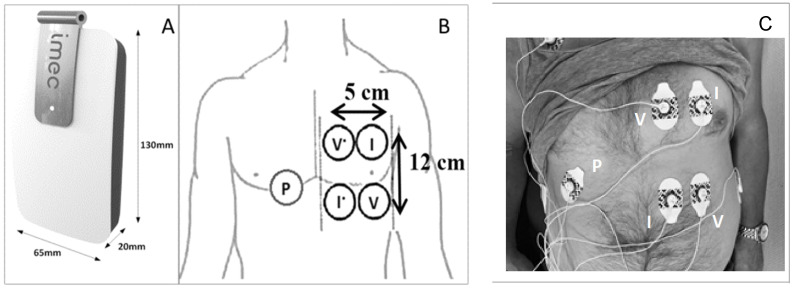
The wearable device (**A**), schematic presentation of the thoracic electrodes (**B**), and attachment of the electrodes and cables on the thoracic region of a study patient (**C**). I current, P bias polar, V voltage.

Thoracic bioimpedance was measured by a wearable device developed by imec the Netherlands (Eindhoven, the Netherlands) [21]. Technical information has been published previously [5]. The electrodes used to perform the thoracic bioimpedance were attached on the patient’s left chest (or right when the vascular access catheter was located on the left side) before the first dialysis session and removed after the second session to eliminate changes in electrode position (Figure 2). The device itself was attached by cables to the electrodes before the start of dialysis and removed at the end of each session. Measurements during hemodialysis were taken in the supine position. During the home measurements, patients were asked to lay in the supine position for 20 min, mimicking their position during hemodialysis. The software in the wearable device was programmed to render bioimpedance data at nine different frequencies (i.e., 8, 10, 13, 16, 20, 26, 40, 80, 160 kHz), every ten minutes. By applying the alternating current at this frequency range, most important physiological information can be obtained. Indeed, at frequencies higher than 200 kHz, more noise is to be expected in the bioimpedance signal [22]. As such, this research device has a limited frequency of up to 160 kHz.

### 2.3. Bioimpedance Signal Processing

Bioimpedance measurements are dependent on the posture and the level of movement of the patient [23]. Therefore, only bioimpedance data recorded under the same posture and during periods of low movement intensity were selected for analysis. To do so, the accelerometer data of the thoracic device were used to derive the static posture and the dynamic movement of the subject at the time of measurement. Secondly, as the resistance component (a measure of voltage divided by current in a resistor) of bioimpedance represents the total body water volume, and as the volume changes in dialysis patients are of main interest in our research, most changes are expected in the resistance component. Therefore, solely the resistance data were selected for further analysis. Thirdly, outliers of the bioimpedance data were detected and removed. Therefore, data were normalized to the median of each session to eliminate inter-subject variability. The normalized values were computed by expressing each measurement as percentage of the median of that session. Outliers in the normalized data were defined as data points that were outside the normal range of the measurements (mean of all data normalized to the median ± 3 × standard deviation). 

### 2.4. Statistical Analysis

Baseline characteristics of the population are expressed as mean ± standard deviation if normally distributed and median [25th–75th percentile] for skewed data. Dichotomous data are expressed as the absolute number and frequency (%). 

The data were approached in three methods, which are listed here and more specified below (Figure 1). First, a global description of the evolution of the resistance over time, at all frequencies, was performed. Second, to investigate the time pattern of semi-continuous and multi-frequency measurements, linear slopes were fitted to a selection of measurements during the first 180 min of the dialysis sessions, specifically at the lowest and the highest frequency of the resistance data. Third, to integrate all semi-continuous measurements and the home measurement, a statistical model was built, taking missing values and the hierarchical structure of the data into account, w*i*th a focus on 8 and 160 kHz.

#### 2.4.1. Descriptive Approach: The Global Evolution during and In-Between the Hemodialysis Sessions

The evolution of the resistance over time, at all frequencies, is described as mean ± standard deviation. Hereto, four intervals were considered: from pre- to end-dialysis session 1 (expressed in hours as ∆T0–T4), from end-dialysis session 1 to the home measurement (expressed in hours as ∆T4–T24), from the home measurement to pre-dialysis session 2 (expressed in hours as ∆T24–T48), and from pre- to end-dialysis session 2 (expressed in hours as ∆T48–T52). The evolution of resistance was visually compared to the evolution of weight. Furthermore, to zoom in on the differences w*i*thin each interval, the number of increasing or decreasing signals per interval was described.

#### 2.4.2. Selective Approach: Comparing the Slopes of 8 and 160 kHz during the First 180 min of Hemodialysis Based on Single-Point Measurements

During hemodialysis, visual inspection of the average trend of the semi-continuous resistance data suggested a different time pattern between the frequencies, which was most clear between 8 and 160 kHz (Supplementary Figure S2). To further explore these time patterns, the resistance data were selectively approached as single-point measurements. To fit a slope and explore the time patterns, the most valuable single-point measurements during hemodialysis were determined. For this, each dialysis session was divided into two parts, based on four different cut-off points according to the visual inspection: 30, 50, 90, and 100 min after the start of dialysis. A selection of measurements up to 180 min after the start of dialysis was considered to avoid extrapolation since only three patients completed all 24 measurements during the 240 min of session 1, and five patients during session 2. For each part (before and after the cut-off point) and each frequency (focus on 8 and 160 kHz), the mean slope of the resistance of all sessions was calculated, ignoring the correlation between two sessions of a single patient and not considering the home measurement in the analysis. For example, the slope *before* 30 min is based on the single-point measurements at 0 and 30 min after the start of dialysis, and the slope *after* 30 min is based on the single-point measurements at 30 and 180 min after the start of dialysis. Thereafter, the slope of the part *before* the cut-off point was compared to the slope of the part *after* the cut-off point by a paired *t*-test. The assumptions (normality and equal variance) underlying the paired *t*-test were checked. The latter was verified by the Brown–Forsyth test. A significant difference between the average slope *before* and *after* the cut-off point could indicate a non-linear time pattern of the resistance signal at a specific frequency during dialysis. Similarly, to investigate the added value of multi-frequency against single-frequency measurements, the slope of the part before the cut-off point at 8 kHz was compared to that at 160 kHz, and likewise for the slope of the part after the cut-off point. A significant difference between the slope of 8 and 160 kHz could advocate multi-frequency instead of single-frequency measurements.

#### 2.4.3. Integrated Approach: Analyzing Semi-Continuous Measurements during Hemodialysis and the Interdialytic Measurement

To integrate all measurements at frequencies 8 and 160 kHz, a statistical model was built incorporating the different dialysis sessions up until 240 min after the start of dialysis as well as the home measurement that was performed. The linear mixed model takes the following form:

(1) $$\begin{array}{c} Y_{ij}=\beta_{0}+(\beta_{1}+u_{1i})S_{1i}+(\beta_{2}+u_{2i})S_{3i}+\beta_{3}F_{8i}+(\beta_{4}+u_{3i})S_{1i}t_{ij}+(\beta_{5}\\ \;\;\;\;\;\;+u_{4i})S_{3i}t_{ij}+\beta_{6}S_{1i}t_{ij}^{2}+\beta_{7}S_{3i}t_{ij}^{2}+\beta_{8}F_{8i}t_{ij}+\beta_{9}F_{8i}t_{ij}^{2}+\varepsilon_{ij} \end{array}$$

where $S_{1i}$ is a dummy variable for the first dialysis session and $S_{3i}$ is a dummy variable for the second dialysis session (Table 1). Consequently, the intercept comprises the home measurement. $F_{8i}$ is 1 if the bioimpedance measurement is taken at a frequency of 8 kHz; if the frequency is 160 kHz, then $F_{8i}$ takes the value 0. $t_{ij}$ represents the time that ranges from 0 to 24, i.e., the dialysis time in minutes divided by 10. Two subject-specific intercepts ($u_{1}$, $u_{2}$) and slopes ($u_{3},\;u_{4}$) were added to the model—one for each dialysis session. $Y_{ij}$ represents the bioimpedance signal for patient *i* at time point *j*. The error term $\varepsilon_{ij}$ is i.i.d. $N\left(0,\sigma^{2}\right)$ and the random effects $u_{1}$, $u_{2}$, $u_{3}$, $u_{4}$ follow a N(0,D), w*i*th D being an unstructured variance–covariance matrix. Comparing evolutions between sessions or frequencies, as well as comparing the starting point and endpoint of sessions, was performed through the construction of linear combinations of the parameters in the linear mixed model, also referred to as contrasts. 

A significance level of 0.05 was used for all tests. Statistical software R version 4.0.4 (R Foundation for Statistical Computing, Vienna, Austria), and SAS 9.4 (SAS Institute Inc., Cary, NC, USA) were used to analyze the data. 

## 3. Results

### 3.1. Baseline Characteristics

The initial cohort consisted of 68 subjects who were measured during three consecutive days, including two dialysis sessions and an interdialytic day. The clinical characteristics of the study participants are represented in Table 2.

### 3.2. Bioimpedance Data Quality

**Figure 3:**
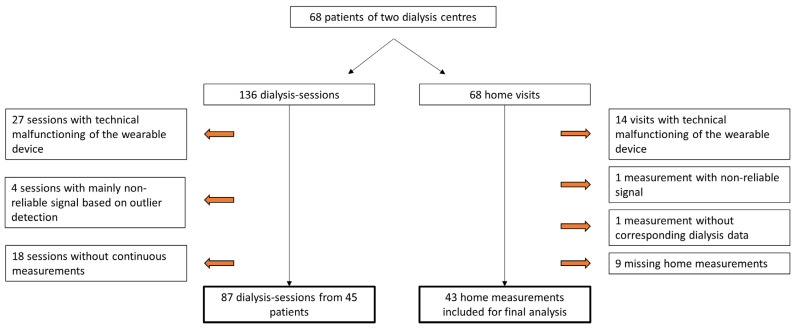
Flow chart of the study process indicating technical malfunctioning of the device, outlier detection, and missing measurements.

The flow chart in Figure 3 describes the in- and exclusion procedure of the data. Due to technical impediments, 19.9% (27/136) of the dialysis sessions and 20.6% (14/68) of the home measurements could not be executed. Semi-continuous measurements during two dialysis sessions at nine frequencies resulted in a total of 17.452 bioimpedance signals in the study population. Outlier detection marked 1.7% (289/17.452) of the measurements during dialysis as non-reliable. An example of the outlier detection for the measurements at 160 kHz during the first dialysis session is represented in Supplementary Figure S1. In-between the two dialysis sessions, patients were visited at home and similar measurements were performed. In 2.3% (9/369) of the home measurements, the device gave a non-reliable signal based on the outlier detection.

### 3.3. Descriptive Characteristics of the Bioimpedance Signal over Time

Semi-continuous measurements visualised the immediate changes in resistance occurring from the start of dialysis (Supplementary Figure S2). These changes seem more pronounced in the low frequencies compared to the high frequencies. 

**Figure 4:**
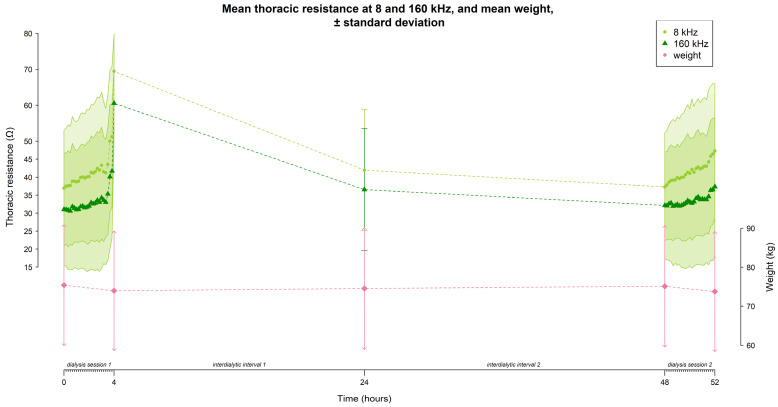
The evolution of thoracic resistance (Ω) w*i*th a focus on 8 and 160 kHz, and weight (kg) over time (hours) throughout the study (i.e., dialysis session 1 from 0–4 h, home measurement at 24 h, and dialysis session 2 from 48–52 h). Every time point represents the mean data from all subjects (n = 45); standard deviations are displayed as the grey shaded area.

The mean resistance at 8 and 160 kHz in function of time is represented in Figure 4 (for all frequencies see Supplementary Figure S2). On average, the thoracic resistance increased over time during fluid removal by hemodialysis and decreased during the interdialytic interval which is characterized by fluid gain due to food and beverage intake.

At 8 kHz, mean thoracic resistance increased from 36.9 ± 16.1 Ω to 69.5 ± 10.4 Ω (∆ 32.6 Ω) during session 1, decreased to 41.9 ± 16.8 Ω (∆ −27.6 Ω) at the home-visit, and decreased further towards the start of session 2 to 37.3 ± 14.9 Ω (∆ −4.6 Ω), before increasing again to 47.3 ± 18.7 Ω (∆ 10 Ω) during session 2 (Figure 4).

Likewise, at 160 kHz, mean thoracic resistance increased from 31.1 ± 15.7 Ω to 60.6 ± 9.9 Ω (∆ 29.5 Ω) during session 1, decreased to 36.6 ± 16.9 Ω (∆ −24 Ω) at the home-visit, and decreased further towards the start of session 2 to 32.2 ± 14.9 Ω (∆ −4.4 Ω), before increasing again during session 2 to 37.3 ± 19.2 Ω (∆ 5.1 Ω) (Figure 4).

The mean weight of the study population evolved from 75.4 ± 15.3 kg to 73.9 ± 15.2 kg (∆ −1.5 kg) during session 1, increased to 74.5 ± 15.5 kg (∆ 0.6 kg) during the home-visit, and increased further towards the start of session 2 to 75.1 ± 15.4 kg (∆ 0.6 kg), before decreasing again during session 2 to 73.8 ± 15.2 kg (∆ −1.3 kg) (Figure 4).

**Figure 5:**
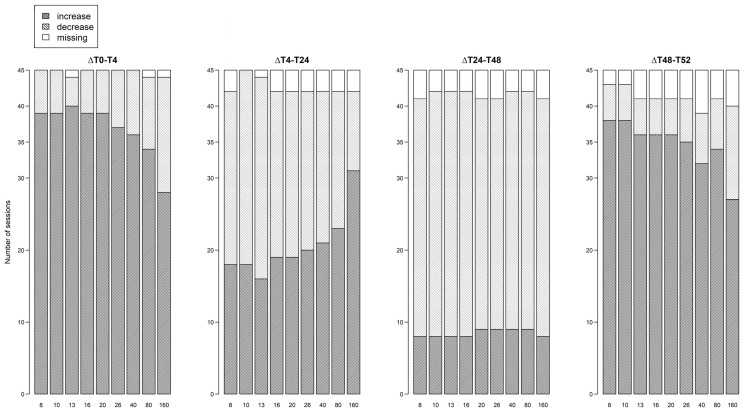
Number of dialysis sessions w*i*th increasing or decreasing thoracic resistance for each frequency, per interval.

**Figure 6:**
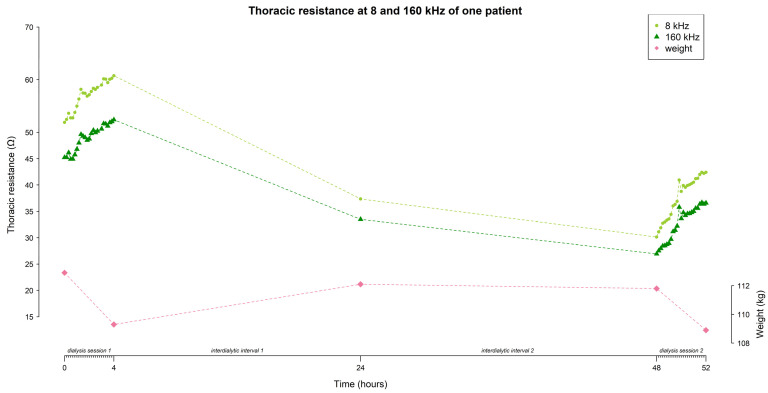
A subject-specific evolution of thoracic resistance (Ω) at 8 and 160 kHz, and weight (kg) over time, demonstrating **an increase** in resistance during both dialysis sessions, and a decrease during the interdialytic interval.

**Figure 7:**
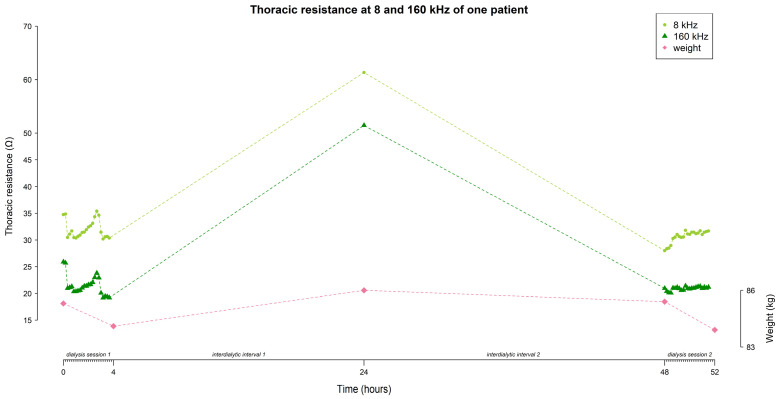
A subject-specific evolution of thoracic resistance (Ω) at 8 and 160 kHz, and weight (kg) over time, demonstrating a **decrease** in resistance during dialysis session 1, an increase during the first interdialytic interval, a decrease during the second interdialytic interval, and an increase during dialysis session 2.

During hemodialysis (∆T0–T4 and ∆T48–T52), the percentage of subjects w*i*th an increasing resistance signal was higher at 8 kHz compared to 160 kHz (session 1: 86.7% vs. 64.4%; session 2: 84.4% vs. 62.2% respectively, Figure 5). Vice versa, the percentage of subjects w*i*th a decreasing resistance signal during dialysis was lower at 8 kHz compared to 160 kHz (session 1: 13.3% vs. 35.6%; session 2: 11.1% vs. 28.9% respectively, Figure 5). To study the subject-specific behaviour of the increasing and decreasing resistance signals at 8 kHz and 160 kHz, individual profiles were visualised as seen in Figure 6 and Figure 7.

During the first interdialytic interval (∆T4–T24), half of the subjects showed a decreasing signal at 8 kHz (53.3%), while at 160 kHz the majority of subjects still showed an increasing signal (68.9%) (Figure 5).

During the second interdialytic interval (∆T24–T48), the decrease in resistance was present in most of the subjects both at 8 kHz (73.3%), and 160 kHz (73.3%) (Figure 5).

### 3.4. Selective Analysis of the Slopes

**Figure 8:**
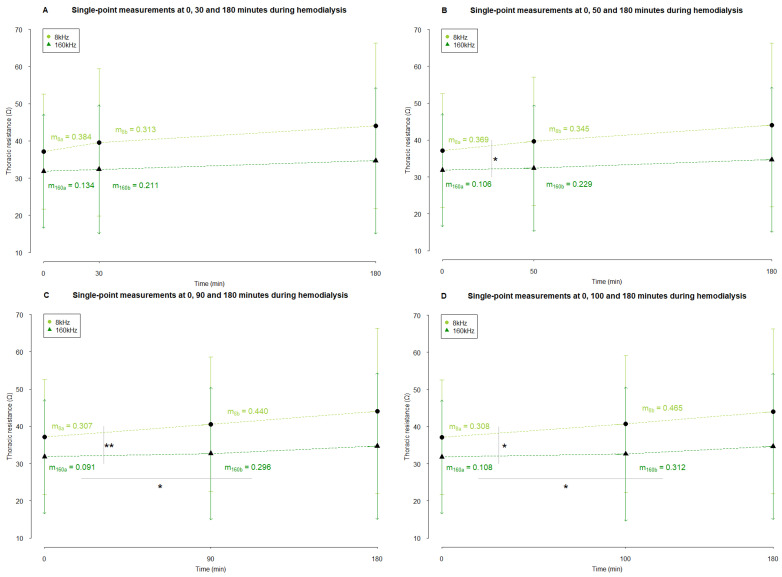
The average slopes of thoracic resistance during hemodialysis based on single-point measurements. Resistance measurements are represented in Ω as mean ± standard deviation (arrowhead as a straight line for 8 kHz and a simple arrowhead for 160 kHz). The cut-off that divided a dialysis session was set at 30 (**A**), 50 (**B**), 90 (**C**), and 100 (**D**) minutes after the start of dialysis. The slopes were compared using a paired sample *t*-test. * and ** indicate *p* values < 0.05 and <0.01, respectively. Abbreviations: m, slope. m_a_ and m_b_ indicate the slope of the part *before* and *after* the cut-off point, respectively.

The analysis of the average resistance slopes compiled by a cut-off at 30 min revealed no significant difference between the slope *before* and *after* the cut-off point, nor between frequencies at 8 and 160 kHz (Figure 8A).

There was no statistical difference between the part *before* and *after* the cut-off of 50 min after the start of dialysis (Figure 8B). The slope of the resistance at 8 kHz *before* this cut-off point was significantly different from the slope at 160 kHz (*p =* 0.042) (Figure 8B). *After* the cut-off point of 50 min, there was no difference between the slopes of both frequencies.

When the cut-off was considered at 90 min, no difference in the resistance slopes at 8 kHz between *before* and *after* the cut-off point was seen (Figure 8C). However, at 160 kHz, there was a statistical difference (*p =* 0.013, Figure 8C). Before the cut-off of 90 min, the resistance slope at 8 kHz was significantly different from the one at 160 kHz (*p =* 0.007, Figure 8C). After this cut-off point, there was no difference anymore between the slopes of both frequencies.

Setting the cut-off at 100 min after the start of dialysis revealed equivalent results as setting the cut-off at 90 min (Figure 8D), Whereas no difference was seen between the resistance slopes *before* and *after* the cut-off point at 8 kHz, the resistance slopes at 160 kHz showed a significantly different trend (*p =* 0.015, Figure 8D). Before this cut-off point, the slope of resistance at 8 kHz was statistically different from the slope at 160 kHz (*p =* 0.011, Figure 8D). After 100 min, no difference between the slopes of frequency 8 and 160 kHz was seen.

### 3.5. An Integrated Approach of Analyzing Semi-Continuous Measurements

The covariance parameter estimates of the mixed model are shown in Table 3. The subject-specific intercepts of both hemodialysis sessions were very variable since both had a high variance [244.98 (standard error (SE) = 52.46) and 241.93 (SE = 52.15), respectively, Table 3]. In other words, the between-patient variability of the thoracic resistance before the start of dialysis was high. 

In addition, a high positive covariance between both random intercepts was noticed (218.98 (SE = 49.58), Table 3). This means that if a patient has a high Ω value at the start of the first hemodialysis session, that patient will most likely have a high starting value in the second hemodialysis session. Comparison of the start of both dialysis sessions (same results for 8 kHz and 160 kHz) resulted in an estimated difference of −0.12 Ω (95% CI: “−2.29”–“2.05”, *p =* 0.912, Table 4).

Furthermore, most between-patient variability was captured in the random intercepts [244.98 (SE = 52.46) in the first session and 241.93 (SE = 52.15) in the second session, Table 3], which were much higher than the random slopes [0.13 (SE = 0.03) and 0.07 (SE = 0.02), respectively, Table 3]. Hence, the variability in resistance between patients is mainly captured by differences in the starting value of a dialysis session rather than differences during hemodialysis. Even though the variances of the random slopes are small, it still suggests that patients tend to differ w*i*th respect to the evolution of their resistance over time.

Next, the positive covariance between both random slopes 0.08 (SE = 0.02) indicates that a higher slope in the first session resulted in a higher slope in the second session (Table 3). Likewise, a positive covariance between the random intercepts and slopes was seen (Table 3). This indicates that patients w*i*th a higher intercept have a bigger slope compared to those w*i*th a lower intercept.

Finally, the w*i*thin-subject variation 12.66 (SE = 0.29) is clearly lower when compared to the between-subject variation 244.98 (SE = 52.47) in the first session and 241.93 (SE = 52.15) in the second session, (Table 3), which is about 20 times smaller.

The influence of *frequency*, *session,* and *time* on resistance is represented in Table 4 and Table 5.

*Frequency*—At 8 kHz, the home measurement was significantly different compared to the home measurement at 160 kHz (6.212 Ω (SE = 0.289), *p* < 0.0001, Table 5).

*Session*—Both sessions started at a significantly different resistance at 160 kHz compared to the home measurement [−5.401 Ω (SE = 2.376), *p* = 0.023 for the first session and −5.279 Ω (SE = 2.371), *p =* 0.026 for the second session, Table 5] The evolution of the resistance signal during session 1 was not significantly different than the evolution during session 2 (*p =* 0.943).

**Figure 9:**
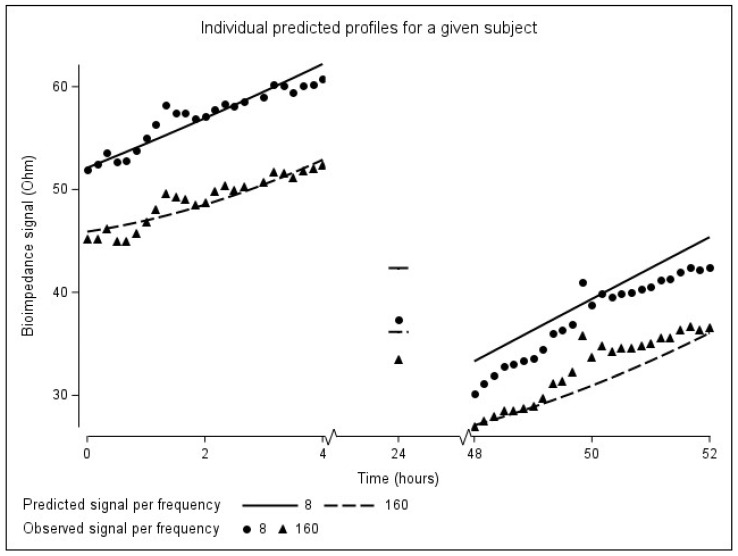
Individual predicted evolution over time for a given patient.

*Time*—The evolution of the resistance signal at 8 kHz over time was statistically different from the evolution of the resistance signal at 160 kHz (*p* < 0.0001). More specifically, to represent the evolution of the resistance over time at 160 kHz, a quadratic term for the first session is needed (*p* = 0.025, Table 5), while the evolution in the second dialysis session can be represented w*i*th just a straight line (*p* = 0.089, Table 5). An individual prediction profile based on the integrated model is shown in Figure 9.

By including the home measurements into the integrated model, an impression could be made of the interdialytic changes in resistance. At 8 kHz, comparison of the end of session 1 w*i*th the home measurement resulted in an average difference in resistance of 2.27 Ω (95% CI: −3.98–8.51, *p* = 0.477, Table 4). At 160 kHz, comparison of the end of session 1 w*i*th the home measurement results in an average difference in resistance of −0.84 Ω (95% CI: −7.09–5.41, *p =* 0.793, Table 4).

## 4. Discussion

This paper shows the feasibility of semi-continuous thoracic bioimpedance measurements by a wearable device w*i*th a multi-frequency electrical current, both during the course of 4 h dialysis sessions, and during the interdialytic interval at home. By measuring thoracic bioimpedance longitudinally and at multi-frequency, different time patterns *w*i*thin* and *between* frequencies appeared, which have several clinical implications.

### 4.1. Technical Feasibility of the Wearable Device

According to the applied outlier detection method, a small percentage of all resistance measurements was labelled as non-reliable. This underlines the capacity of the wearable device to record reliable signals in a semi-continuous way during fluid changes. However, some improvements should be considered. The relatively high number of excluded sessions due to technical problems points out the importance of further optimization of the device. Furthermore, the device that is currently used requests to apply the same electrode configuration for each measurement. Although this was captured by a fixed electrode configuration from the start until the end of this study, the preliminary device as such is not yet suitable for clinical practice. Moreover, its rather large size, the use of cables and multiple electrodes may hinder patients during their daily life activities. Taking the large number of reliable signals into account, together w*i*th the aforementioned limitations of the current device, these observations should encourage the further development of a smaller device (i.e., a patch).

### 4.2. Clinical/Pathophysiological Feasibility 

This feasibility study reveals several important results. First, single-point bioimpedance measurements (1) show a global increasing resistance towards the end of hemodialysis, and a decreasing resistance during the interdialytic interval, (2) suggest a linear time pattern in the resistance at 8 kHz, and a non-linear time pattern in the resistance at 160 kHz, and (3) detect a different time pattern between low- versus high-frequency resistance from 50 min after the start of dialysis on. Second, by measuring bioimpedance semi-continuously, (1) changes over time are detected immediately after the start of hemodialysis, (2) a prediction model could be built, by which the linear time pattern at 8 kHz and a quadratic trend at 160 kHz in the first dialysis session were confirmed, and (3) changes during the interdialytic interval can be interpreted more accurately through the prediction model compared to the single-point measurements. Third, measuring bioimpedance at multiple frequencies reveal that its increase during hemodialysis and decrease during the interdialytic interval is more pronounced in the lower frequencies compared to the higher frequencies. More specifically, the slow increase of the resistance at 160 kHz during hemodialysis even resonates during the first interdialytic interval.

All these observations can mainly be explained pathophysiological by volume changes *in* and *between* body compartments. Both extra- and intracellular volumes are important compartments to study. Whereas an ineffective mobilization from the intracellular volume and the interstitial space (as part of the extracellular volume) can lead to reduced plasma volume and hypotension [24,25], an expansion of extracellular volume leads to hypervolemia and hypertension, and alternations of intracellular volumes impairs many cellular functions [24]. Over the long term, the ratio of extracellular/intracellular volume is associated w*i*th malnutrition and aging [26]. In the theory of bioimpedance, it is known that signals at lower frequencies represent the extracellular volume, whereas higher frequencies additionally take the intracellular volume into account [1]. As such, changes in the extracellular volume influence both low- and high-frequency signals. Subsequently, an increase in resistance at all frequencies indicates the loss of extracellular volume, and vice versa, as expected according to the principles of fluid extraction during a hemodialysis treatment [27]. On the other hand, when interpreting changes in high-frequency resistance, additional changes in intracellular volume must be taken into account. Moreover, bioimpedance at high frequency (>50 kHz) [28] reflects blood pressure changes through changes in the cross-sectional area of the targeted arteria [15,29,30,31].

#### 4.2.1. Semi-Continuous and Multi-Frequency Measurements Enable the Interpretation of Fluid Dynamics during Hemodialysis

During hemodialysis, the immediate resistance changes after the start of dialysis suggest an involvement of volume changes in the central compartment. However, at this time minimal ultrafiltration has been applied. An additional contribution of a decreasing blood pressure during the first 30 min of hemodialysis due to nitric oxide generation may further explain this finding [15,25,30].

Next, the differences *w*i*thin* and *between* bioimpedance frequencies became clear by measuring semi-continuously and at multi-frequency. *W*i*thin* 8 and 160 kHz, a respectively linear versus non-linear time trend could be extracted from the mixed model. The linear relationship between the resistance signal at 8 kHz and time implies a constant rate of change. Previously, it has been shown that thoracic bioimpedance signals at low frequencies correlate very strongly w*i*th ultrafiltration volume [5,18]. The removal of the ultrafiltration volume at a constant rate may explain the linear increase in the resistance signal at 8 kHz. As such, the implication of the linear pattern in the resistance signal at 8 kHz for fluid management can be found in the prediction of fluid changes at a constant rate. More specifically, the more volume that is extracted from the body, the higher its resistance will be. The remaining clinical challenge lies in the detection of a subject-specific reference value which could serve as a critical maximum whereto the ultrafiltration volume can be adjusted. In contrast, the non-linear evolution of the resistance at 160 kHz over time means that this relationship does not have a constant rate of change. This implies that resistance at 160 kHz indeed reflects physiological mechanisms additional to changes in extracellular volume, compared to the resistance at 8 kHz. The process of ultrafiltration during hemodialysis directly lowers extracellular volume and plasma sodium concentration. As plasma sodium concentration is the main inverse determinant of intracellular volume, an indirect increase is to be expected in intracellular volume. Whereas the decrease in extracellular volume is initiated immediately after the start of hemodialysis, the indirect increase in intracellular volume is delayed and occurs towards the end of dialysis [32]. This inertia may explain the non-constant rate of change which is captured w*i*thin the non-linear increase in the resistance at 160 kHz. This feature is emphasized through the implementation of the home measurement, where the majority of the patients show a persisting increase in resistance at 160 kHz during the first interdialytic interval. Hence, when interpreting the non-linear pattern of the resistance signal at 160 kHz, the delayed changes in intracellular volume must be taken into account.

Moreover, by measuring bioimpedance at multi-frequency current, the relative difference *between* 8 and 160 kHz could be interpreted. The slower increase over time 160 kHz compared to 8 kHz suggests a fluid gain into the intracellular volume compartment. An intracellular fluid gain during hemodialysis has been described earlier on a whole-body level and in the limbs, and is mainly due to a decrease in plasma sodium concentration, as is the case in our study population [32,33,34,35,36]. Jain et al. focused on the thoracic segment of five patients, and found a rather decreasing trend in estimations of intracellular volume of the trunk during hemodialysis [37,38]. Anand et al. described a global increasing thoracic bioimpedance signal during hemodialysis [16]. As the frequency they used is not reported, no conclusions can be made on changes in volume compartments.

These results point out the potential of thoracic bioimpedance to serve as a hemodynamic application during hemodialysis. By interpreting fluid dynamics in the light of semi-continuous and multi-frequency bioimpedance recordings, predictions on blood pressure could be made in order to avoid hypotensive episodes or persistent post-dialytic hypervolemia.

#### 4.2.2. Multi-frequency Bioimpedance Measurements during the Interdialytic Interval Provide Valuable Information on Fluid Gain

Considering the home measurements, a significantly higher resistance was measured at home compared to the values at the start of both sessions, and both at low and high frequency. When compared to the measurements taken at the end of the first dialysis session, an average decrease in resistance at all frequencies is observed during the first interdialytic interval. This decrease was more distinct in the lower frequencies compared to the higher frequencies. Indeed, from the mixed model, an average 2.27 Ω decrease in 8 kHz could be calculated versus a small increase of 0.84 Ω in 160 kHz (Table 4) on average. As explained above, the larger decrease in resistance at 8 kHz could indicate a fluid gain in extracellular volume and the milder decrease at 160 kHz implies an additional fluid loss in intracellular volume caused by an increase in plasma sodium concentration and plasma volume due to food and fluid intake from the patient at home [37]. Additionally, our results showed a larger decrease in resistance during the first interdialytic interval compared to the second interdialytic interval. This could imply that the fluid gain in our study population was larger during the first interdialytic interval. However, some caution has to be taken here. Given the large standard deviation of the measurements during the last hour of dialysis, due to missing values, the mean resistance value may not accurately represent the population mean. In addition, the weight gain during both the interdialytic intervals was the same (0.6 kg). The integrated approach, which takes missing values into account, found on average a larger decrease in resistance during the second interdialytic interval. These findings indicate that by remote monitoring of thoracic bioimpedance, in preference semi-continuously, a more accurate approach to fluid gain can be obtained compared to a weight-based approach. As such, this technology could serve as an instant feedback monitoring system on fluid intake for the hemodialysis patient. Similarly, the home-hemodialysis population could benefit from the thoracic bioimpedance system to create a personalized treatment scheme and apply hemodialysis based on critical minimum values.

### 4.3. Personalized Feasibility

In addition to the above-discussed findings, the mixed model used in the integrated approach established subject-specific results. First, there is a high between-subject variability in resistance at the start of both dialysis sessions. This can be mainly explained by subject-specific differences in body composition (i.e., fluid volume, muscle- and fat mass), and plasma concentration of electrolytes between patients. Furthermore, the small w*i*thin-subject variability in resistance at the start of both dialysis sessions suggest that the measurements made by the wearable device are reliable. These results were achieved by a consistent electrode configuration and body position of the patients between both sessions. This confirms the results of our previous work, where thoracic resistance was moderately reproducible between two dialysis sessions [18]. These results strongly suggest that patients have a subject-specific start-point of thoracic bioimpedance. Consequently, a personalized interpretation of the bioimpedance data instead of an average approach is warranted. Although the variances for the random slopes of both sessions were small, it still suggests that patients do differ w*i*th respect to their evolutions over time, as is clearly demonstrated in the individual graphs (Figure 6 and Figure 7).

### 4.4. Limitations

Some limitations have to be mentioned in this study. First, towards the end of the hemodialysis sessions, the standard deviation of the bioimpedance measurement is high because of missing values. As patients did not want to postpone the termination of their treatment, a disconnection of the bioimpedance device before the end of dialysis was unavoidable. However, by analyzing the data by mixed modelling, these missing values could be taken into account and the results could be interpreted in the most feasible way. Second, it should be kept in mind that the assumptions of the linear mixed model were violated. As such, some caution is warranted in the interpretation of the results. Third, the purpose of this study was to obtain a more detailed look into the longitudinal time pattern of the bioimpedance signal. Although this was practically feasible during hemodialysis, this was not the case during the interdialytic interval due to the life-span of the battery and the inconvenience for the patient to take care for the device at home. Therefore, we performed a home visit, which resulted in a 20 min measurement. In future research, this limitation could be addressed by providing the bioimpedance technique into a patch.

### 4.5. Clinical Implementation and Future Perspectives

The results obtained by this feasibility study could benefit the population w*i*th end-stage renal disease in its broadest sense. Patients treated w*i*th hemodialysis, both in-center and at home, could be equipped w*i*th a wearable device that semi-continuously measures multi-frequency thoracic bioimpedance. The device could function as a remote monitoring tool by interpreting certain trends in the signal, obtained by semi-continuous multi-frequency recordings. For example, dedicated clinicians could be warned when a patient reaches a critical maximum thoracic resistance and lower the ultrafiltration rate in order to avoid intradialytic hypotension. From the patient’s perspective, the introduction of a wearable bioimpedance device could modulate their personalized participation in their own hemodialysis treatment. For example, patients could be instructed to schedule a dialysis-session when the thoracic resistance reaches a critical minimum in order to avoid fluid overload. In addition, the home-monitoring function of the device could be extended to other patient groups who also often suffer from frequent fluid changes like patients w*i*th non-dialysis end-stage renal disease or chronic heart failure.

This work reaches out to several future perspectives. In the near future, the production of a wearable device that is easy-to-use for patients should be continued. Hereby, some technical challenges will have to be faced. W*i*th respect to the hardware, the introduction of skin-sensors instead of cables and the further miniaturization of the device should be achieved. In fact, similar to the continuous rhythm monitoring of heart failure patients, the device could be designed as an implantable form. W*i*th respect to the software, a wireless connecting platform from where the research- or nursing staff can detect changes in bioimpedance trends could be created. Furthermore, the extension of the frequency range up to 1000 kHz would lead to the opportunity to create, interpret and model the Cole–Cole plots in order to provide an informative and predictive overview of the bioimpedance signals.

## 5. Conclusions

This study demonstrates the feasibility of measuring thoracic multi-frequency bioimpedance semi-continuously in a hemodialysis population. Longitudinal bioimpedance data provide a broader and profounder knowledge on fluid dynamics compared to single-point and single-frequency measurements. The key findings are (1) an increase in resistance during hemodialysis due to fluid loss and a decrease during the interdialytic interval due to fluid gain, (2) the discovery of an inertia w*i*thin the higher frequencies, possibly related to delayed changes in intracellular volume, (3) patients have a subject-specific start-point of thoracic bioimpedance. Hence, measuring bioimpedance of the thoracic region should be performed by a device equipped w*i*th a multi-frequency electrical current. In the future, this innovative tool should be explored as a remote hemodynamic monitoring application in dialysis patients. Real-time access to longitudinal data could initiate novel strategies that can efficiently “close the loop” of fluid management by accurately monitor hemodynamics and guide a constant fine-tuning of ultrafiltration volume during hemodialysis or patients self-adjustment in fluid intake and planning of home-hemodialysis before hypotensive or hypervolemic episodes occur.

## Figures and Tables

**Table 1:** Overview of the parameters in the equation of the linear mixed model.

Abbreviation	Variable
$S_{1i}$	Session 1 for the *i*th patient
$S_{2i}$	Home measurement for the *i*th patient
$S_{3i}$	Session 2 for the *i*th patient
$F_{8i}$	Resistance signals at 8 kHz for the *i*th patient
$F_{160i}$	Resistance signals at 160 kHz for the *i*th patient
$t_{ij}$	*j*th time point for the *i*th patient
$u_{1}$	Subject-specific intercept for session 1
$u_{2}$	Subject-specific intercept for session 2
$u_{3}$	Subject-specific slope for session 1
$u_{4}$	Subject-specific slope for session 2
$Y_{ij}$	Outcome, the bioimpedance signal for patient *i* at time point *j*
$\varepsilon_{ij}$	Error term for patient *i* at time point *j*
*β*	Estimates of the fixed effects

**Table 2:** The clinical characteristics of study participants.

	Total Cohort (*n* = 68)
Age (years)	70.4 ± 13.2
Gender (male)	46 (67.4%)
BMI (kg/m^2^) ^a^	26.3 ± 5.5
Obesity ^b^	13 (24.1%)
Fistula—Hickmann catheter	31 (45.6%)–37 (54.4%)
Kt/V	1.4 ± 0.3
Mean pre-dialysis SBP/DBP (mmHg)	135.7 ± 20.3/66.1 ± 16.4
Mean plasma sodium concentration (mmol/L) - Pre-dialytic - Post-dialytic	138.7 138.3
Dialysis vintage (years)	3.9 ± 3.7
UFV (mL)	1539.7 ± 897.4
Diabetes mellitus	31 (45.6%)
Heart failure ^c^	25 (36.2%)
COPD	6 (11.1%)

^a^ based on target weight; ^b^ based on BMI ≥ 30 kg/m^2^; ^c^ heart failure includes systolic or diastolic dysfunction. Data are represented as mean ± standard deviation or number (percentage) as appropriate. Abbreviations: *COPD* chronic obstructive pulmonary disease; *DBP* diastolic blood pressure; *SBP* systolic blood pressure; *UFV* ultrafiltration volume.

**Table 3:** Covariance parameter estimates and standard errors of the statistical model for the integrated approach.

	Estimate	Standard Error
Variance (Int session 1)	244.98	52.46
Covariance (Int session 1, Int session 2)	218.98	49.58
Variance (Int session 2)	241.93	52.15
Covariance (Int session 1, Slope session 1)	2.56	0.95
Covariance (Int session 2, Slope session 1)	3.1	0.98
Variance (Slope session 1)	0.13	0.03
Covariance (Int session 1, Slope session 2)	2.06	0.74
Covariance (Int session 1, Slope session 2)	2.23	0.74
Covariance (Slope session 1, Slope session 2)	0.08	0.02
Variance (Slope session 2)	0.07	0.02
Residual	12.66	0.29

Abbreviations: *Int* intercept.

**Table 4:** Contrast statistics for the integrated approach. Bold format indicates statistically significant *p* value.

Effect	Frequency	Estimate	Standard Error	*t*-Value	*p* Value	95% Confidence Interval
End session 1 versus home measurement	8	2.27	3.19	0.71	0.477	“−3.98”–“8.51”
	160	−0.84	3.19	−0.26	0.793	“−7.09”–“5.41”
Start session 2 versus home measurement	8	−5.28	2.37	−2.23	**0.026**	“−9.93”–“−0.63”
	160	−5.28	2.37	−2.23	**0.026**	“−9.93”–“−0.63”
Start session 1 versus start session 2	8	−0.12	1.11	−0.11	0.912	“−2.29”–“2.05”
	160	−0.12	1.11	−0.11	0.912	“−2.29”–“2.05”
End session 1 versus end session 2	8	0.21	1.25	0.17	0.864	“−2.23”–“2.66”
	160	0.21	1.25	0.17	0.864	“−2.23”–“2.66”

**Table 5:** Solution for fixed effects of the statistical model for the integrated approach.

Effect	Frequency	Session	Estimate	Standard Error	*t*-Value	*p* Value
Intercept			36.148	0.417	86.67	**<0.0001**
Frequency $\left(F_{8i}\right)$	8		6.212	0.289	21.47	**<0.0001**
Frequency	160		0	-	-	-
Session $\left(S_{1i}\right)$		1	−5.401	2.376	−2.27	**0.023**
Session $\left(S_{3i}\right)$		2	−5.279	2.371	−2.23	**0.026**
Session		Home	0	-	-	-
Time × session ($S_{1i}t_{ij})$		1	0.045	0.079	0.58	0.564
Time × session ($S_{3i}t_{ij})$		2	0.072	0.070	1.04	0.300
Time × session		Home	0	-	-	-
Time × time × session $\left(S_{1i}t_{ij}^{2}\right)$		1	0.006	0.003	2.23	**0.026**
Time × time × session $\left(S_{3i}t_{ij}^{2}\right)$		2	0.004	0.003	1.70	0.089
Time × time × session		home	0	-	-	-
Time × frequency $\left(F_{8i}t_{ij}\right)$	8		0.238	0.063	3.78	**0.0002**
Time × frequency	160		0	-	-	-
Time × time × frequency $\left(F_{8i}t_{ij}^{2}\right)$	8		−0.005	0.003	−1.58	0.114
Time × time × frequency	160		0	-	-	-

## Data Availability

The datasets used or analyzed during the current study are available from the corresponding author on reasonable request.

## Supplementary Materials

The following supporting information can be downloaded at: https://www.mdpi.com/article/10.3390/s24061890/s1, Figure S1: Example of the outlier detection method for measurements during session 1 at 160 kHz frequency. Each line represents the measurements for one patient. Measurements w*i*thin the shaded area were considered as outliers. Figure S2: The evolution of the thoracic resistance (Ohm) over time (minutes) throughout the first 180 min of hemodialysis. Every line represents a different frequency. Every time point represents the mean resistance of all dialysis sessions (n = 87).
