# Supplementary figures and images for: The Feasibility of Semi-Continuous and Multi-Frequency Thoracic Bioimpedance Measurements by a Wearable Device during Fluid Changes in Hemodialysis Patients
Source: Sensors (Basel). 2024 Mar 15;24(6):1890. doi: 10.3390/s24061890 (PMC10976053; doi:10.3390/s24061890)

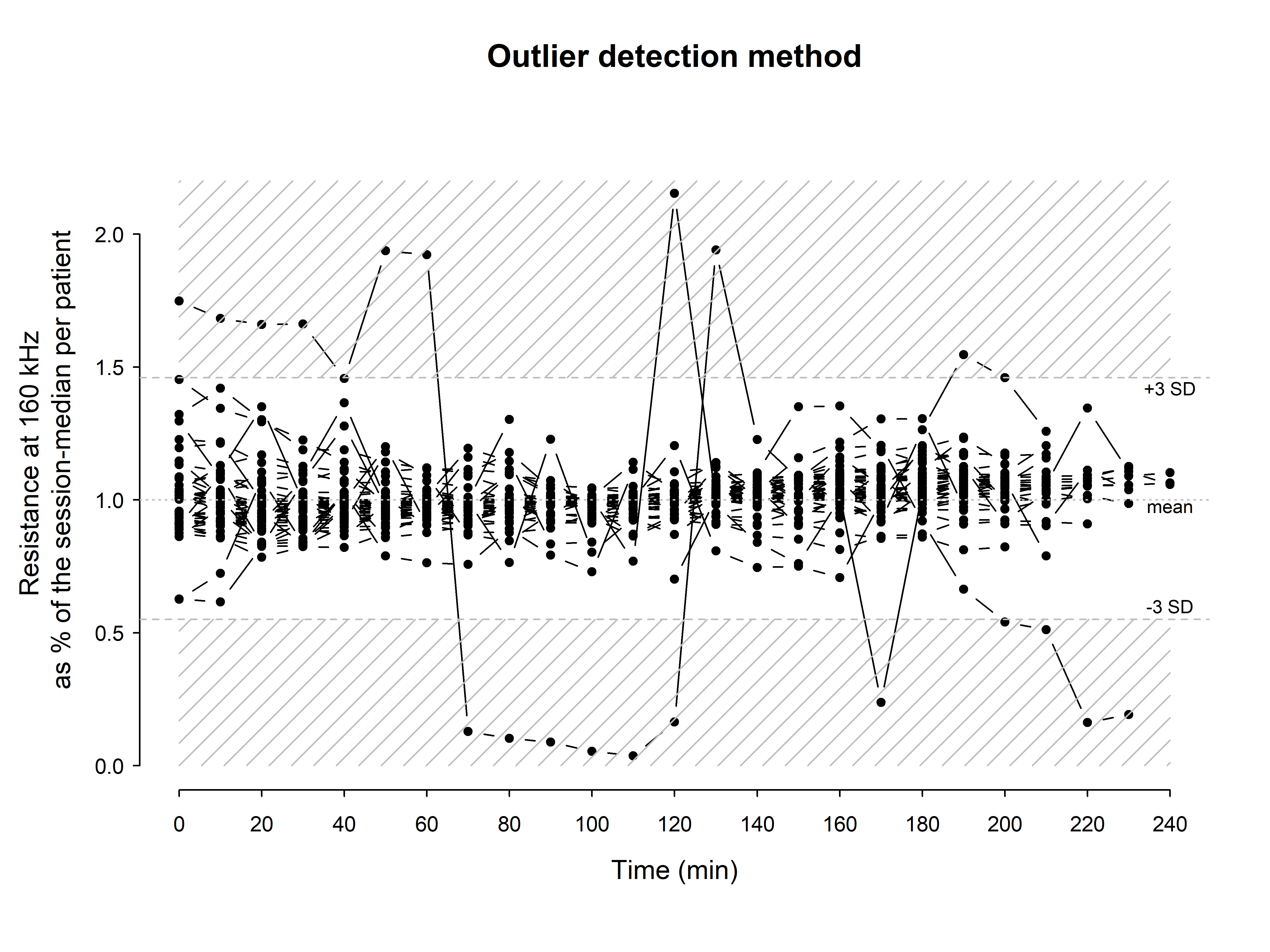

Supplement: Supplementary file 1 [file sensors-24-01890-s001.zip › Figure S1.tiff]

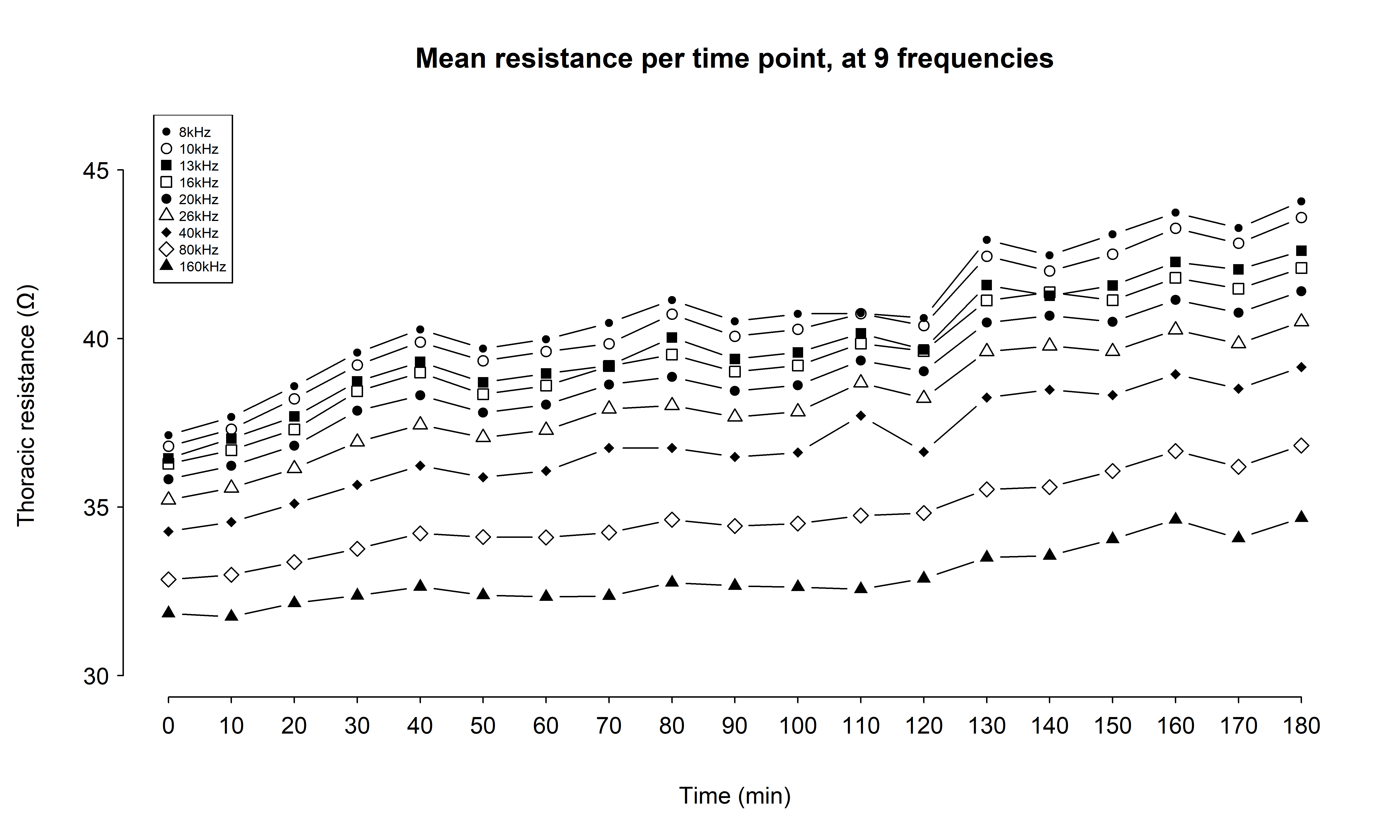

Supplement: Supplementary file 1 [file sensors-24-01890-s001.zip › Figure S2.tiff]
